# Selective Prediction With Long Short-term Memory Using Unit-Wise Batch Standardization for Time Series Health Data Sets: Algorithm Development and Validation

**DOI:** 10.2196/30587

**Published:** 2022-03-15

**Authors:** Borum Nam, Joo Young Kim, In Young Kim, Baek Hwan Cho

**Affiliations:** 1 Department of Electronic Engineering Hanyang University Seoul Republic of Korea; 2 Department of Biomedical Engineering Hanyang University Seoul Republic of Korea; 3 Medical AI Research Center Samsung Medical Center Seoul Republic of Korea

**Keywords:** artificial intelligence, recurrent neural networks, biomedical informatics, computer-aided analysis, mobile phone

## Abstract

**Background:**

In any health care system, both the classification of data and the confidence level of such classifications are important. Therefore, a selective prediction model is required to classify time series health data according to confidence levels of prediction.

**Objective:**

This study aims to develop a method using long short-term memory (LSTM) models with a reject option for time series health data classification.

**Methods:**

An existing selective prediction method was adopted to implement an option for rejecting a classification output in LSTM models. However, a conventional selection function approach to LSTM does not achieve acceptable performance during learning stages. To tackle this problem, we proposed a unit-wise batch standardization that attempts to normalize each hidden unit in LSTM to apply the structural characteristics of LSTM models that concern the selection function.

**Results:**

The ability of our method to approximate the target confidence level was compared by coverage violations for 2 time series of health data sets consisting of human activity and arrhythmia. For both data sets, our approach yielded lower average coverage violations (0.98% and 1.79% for each data set) than those of the conventional approach. In addition, the classification performance when using the reject option was compared with that of other normalization methods. Our method demonstrated superior performance for selective risk (12.63% and 17.82% for each data set), false-positive rates (2.09% and 5.8% for each data set), and false-negative rates (10.58% and 17.24% for each data set).

**Conclusions:**

Our normalization approach can help make selective predictions for time series health data. We expect this technique to enhance the confidence of users in classification systems and improve collaborative efforts between humans and artificial intelligence in the medical field through the use of classification that considers confidence.

## Introduction

### Background

High-performance networks have been used to enhance the quality and convenience of human life since the development of deep learning techniques. Deep learning networks are used in education, aviation, process management, entertainment, agriculture, and robotics. Artificial intelligence (AI) has made significant contributions to a variety of medical applications [1-3]. However, in a clinical setting, the output from AI as an accurate prediction is often insufficient and requires its interpretation for further decisions [4]. As medical AI systems can support efficient and accurate decisions, it is important not only to increase the accuracy of classification in deep learning networks but also to reduce errors, particularly those that can be fatal [5]. In addition, health care data tend to be complex, and neural networks have proven problematic in accurately recognizing patterns in this complexity [6]. The uncertainty of prediction measures the reliability of a prediction and must be considered in fields that require prudent decisions, such as medicine or autonomous driving [7]. Accordingly, in fields where minor errors can cause significant problems, applying a prediction model that can reject predictions when the confidence level is not high enough is helpful. To develop such a deep neural network, a selective prediction [8] method can be applied to use the confidence level in both training and test sessions.

Various biosignal sensors have been developed for human health care applications, and many algorithms have been developed to analyze the data produced by these sensors. Deep learning technologies have performed well when applied to data obtained from health care or medical sensors [9]. Classification models based on a deep neural network or convolutional neural network (CNN) have been used to classify health and medical data. In addition, biosignals and time series data from humans are used in diverse health care systems [10]. In various studies, recurrent neural network (RNN) models have been used to classify health and medical data, especially time series data. Among such models, RNNs have contributed significantly to the classification of time series data. Many studies have used RNN models to classify electronic health records obtained from clinical measurements [11], predict diseases using patient diagnostic histories [12-14], conduct health status analyses using biosignals [15-18], and classify health information from mobile and wearable sensors [19-22]. Previous studies have applied prediction confidence to classify image data, and prediction confidence can be considered for classifying time series health data using RNN models. However, little research has focused on how to use prediction confidence for time series health data.

Considering the specificity of time series health data, a model that can produce results according to the predicted confidence level and uses prediction confidence has the advantage of reducing fatal errors.

The selective prediction model can learn from certain samples that are sufficiently confident in their predictions. This means that such a model can ignore predictions when they are uncertain in training. In addition, the selective prediction model provides a confidence level for each test sample in the inference stage, which can be used as a reference score in a medical situation. In early studies on selective prediction, neural network models with a reject option were used to obtain a specific confidence score from a trained model and as a model threshold to validate performance [23-25]. However, these methods calculate the prediction probability to select samples for training based on a threshold called the prediction confidence score.

Recently, research using the selective prediction model mainly consists of 2 parts. The first is to extract an appropriate prediction confidence score and the second is to make good use of the extracted prediction confidence score for the deep learning model. For extracting the prediction confidence score, methods have been designed in many studies. For example, the softmax response and Monte Carlo (MC) dropout methods use a confidence score from neural networks [26]. The softmax response method extracts a confidence score using maximum softmax values from neural networks, as described in the above methods, whereas an MC dropout estimates a confidence score using statistical approaches. However, MC dropout requires a high computational cost to optimize the problem quickly. Although Bayesian methods [27-29] can produce prediction confidence scores of RNNs [30], they are applicable only for natural language processing, which uses *many-to-many* RNNs with multiple sequence inputs and outputs. However, the predictive models in health care are usually *many-to-one* types that predict class using a health information time series as input, and it is helpful for medical staff to train a *many-to-one* predictive model for time series data that has a selective prediction ability. For a model using the prediction confidence score, a selective prediction model that learned both prediction and selection was developed [31]. On the basis of this method, SelectiveNet [32] has demonstrated potential possibilities for various applications, with the advantage of learning the selection and prediction simultaneously. However, the structure of the selective prediction model using long short-term memory (LSTM) has not been validated in previous studies. Thus, a well-designed selective prediction model for time series data is required.

### Objective

In this study, a selective prediction model using LSTM [33] was implemented to classify time series health data. In particular, we considered a method that incorporates a reject option to control and measure prediction confidence for *many-to-one* classification tasks. As the selection function uses the output of the prediction model as an input, a suitable selection function structure must be devised. Therefore, methods to normalize the selection function were compared to achieve a structure suitable for classifying time series data with LSTM. To validate the LSTM selective prediction performance, we used coverage violations and selective risks for each data set. As high false-positive and false-negative rates can be critical factors in diagnoses, we also present the false-positive and false-negative rates of the LSTM selective prediction model. In summary, the goal of this study is to develop a selective prediction model for health data time series. The contributions of this study are (1) applying the latest selective prediction method with superior performance to classify time series health data using LSTM and (2) presenting the structure of the selection function in the selective prediction model (especially the normalization method) for time series selective prediction.

## Methods

### Selective Prediction

**Figure 1:**
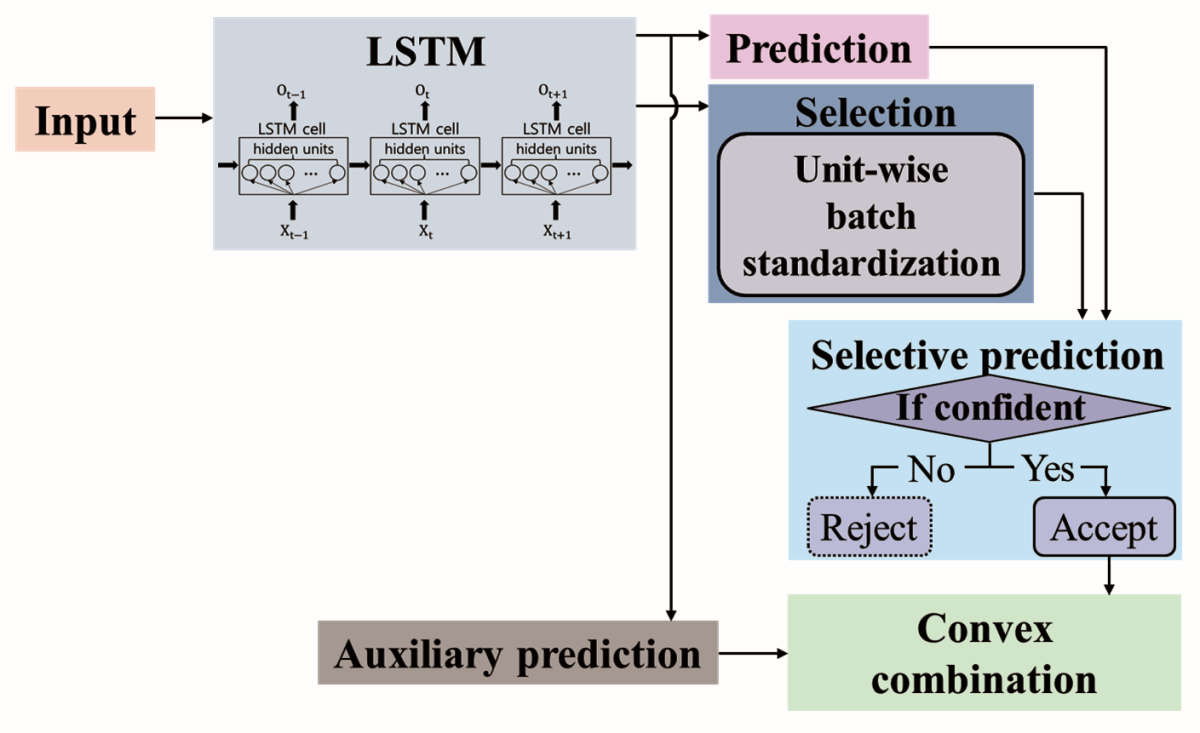
Long short-term memory model structure with a reject option. LSTM: long short-term memory.

We examined the possibility of RNN models with a reject option using SelectiveNet [32], which has superior performance compared with existing selective prediction models. The overall structure of the model was based on the SelectiveNet [32] model with an LSTM; it is divided into selective and auxiliary predictions, as shown in Figure 1. The selective prediction is divided again into two steps: prediction and selection. Prediction involves the results of the LSTM model and the selection part extracts the predicted confidence level of the LSTM model. In this study, we propose unit-wise batch standardization (UBS) as part of the selection function. Selective prediction is performed using both the prediction and selection function results. An auxiliary prediction step using the LSTM prediction result to derive the final result with the selective prediction result was added to enhance prediction performance. As selective prediction is a prediction model using a deep learning model structure, it is optimized by a loss function. The entire model is trained by optimizing the selective prediction and auxiliary prediction steps simultaneously. Further details are provided in the *Optimization* section. LSTM was used for the RNN model for time series data classification.

A selective model was used to implement classification models with the reject option [34]. The selective model (*f, g*) consists of pairing a prediction function *f* and a selection function *g:X→Y {Y|0≤Y≤1}* (*X* is a set of inputs and *Y* is a set of outputs). When the data set is given as 
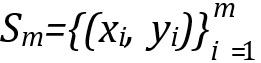
 for supervised learning of the classification model, the empirical risk of prediction function *f* becomes 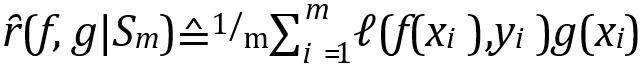. When *τ* is a threshold, *g* acts as a qualifier of *f* and can be expressed as follows:




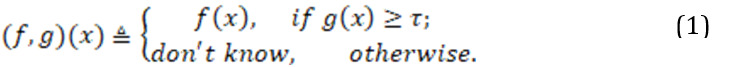




Selective models can be controlled by coverage and risk values. When *E_p_* is the expected probability, and ℓ is the loss function, we can define the coverage and risk as follows:




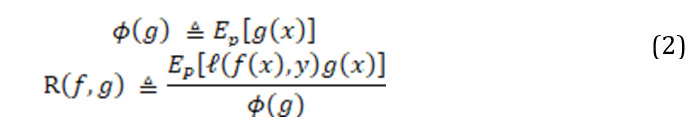




where *g*(*x*) is the prediction confidence score, *ϕ*(*g*) is a coverage value that is the expected value of the prediction confidence scores for training samples, which is correlated with the number of selected samples during training. R(*f, g*) is a selective risk that represents the error rate for predicting the selected samples using selective prediction. The corresponding selective risk for a data set 
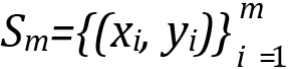
is called the empirical selective risk and is defined as follows:



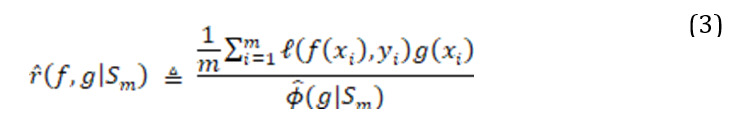



The empirical coverage corresponding to the data set *S_m_* is as follows:



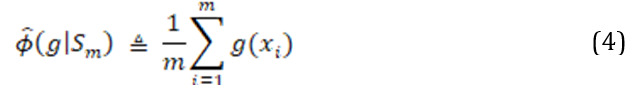



### Optimization

An optimization method was used to constrain coverage and reduce the selective risk [31]. The selective prediction model was optimized by the loss functions in equations 6, 7, and 8. This loss function simultaneously regulates the prediction and selection steps. Hence, the selective prediction was regulated to lower the error rate, which is the selective risk for the selected samples according to the prediction confidence. In addition, the selection step was optimized to select training samples based on the predefined target coverage so that the selection step would reject predictions below the confidence level. The target coverage is a controlling hyperparameter for the model to learn the amount of data to be selected during training. On the basis of this, we trained the model so that the coverage value was as close to the target coverage as possible. The target coverage *c* is in the range 0<*c*≤1. When the parameter set of the selective model (*f, g*) is Θ the optimization of the selective model is as follows:



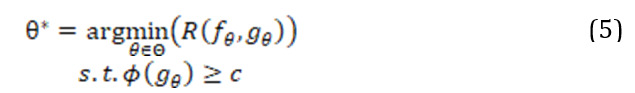



The *f_θ_* and *g_θ_* in the selective prediction were optimized by equation 6. It is necessary to constrain coverage and reduce risk (error) for selective prediction. We used the interior point method for optimization [35]. The following unconstrained objective is used to optimize the selective prediction model for a data set *S_m_*:



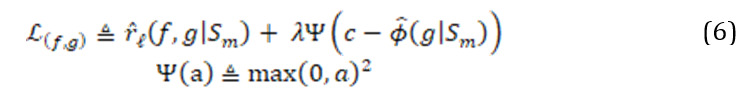



where *c* is the target coverage, and λ is a hyperparameter that controls the coverage constraints. Using equation 6, the selection function *g* is optimized to produce an appropriate prediction confidence score, and the selective prediction is optimized to reduce the selective risk 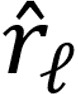. The empirical coverage value 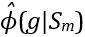 is probabilistically calculated using the selection function. The Ψ allows the coverage value 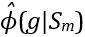
to approximate the target coverage during the training session. The auxiliary classification loss is optimized using the loss function 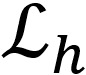. Overall, optimization can be defined using a convex combination expressed by the following equations:



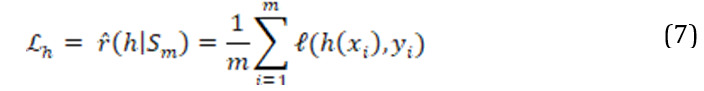





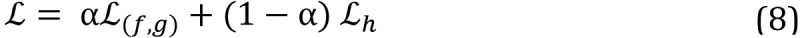



where α is another user-controlled parameter for the weights between the selective and auxiliary predictions.

### UBS Procedure

**Figure 2:**
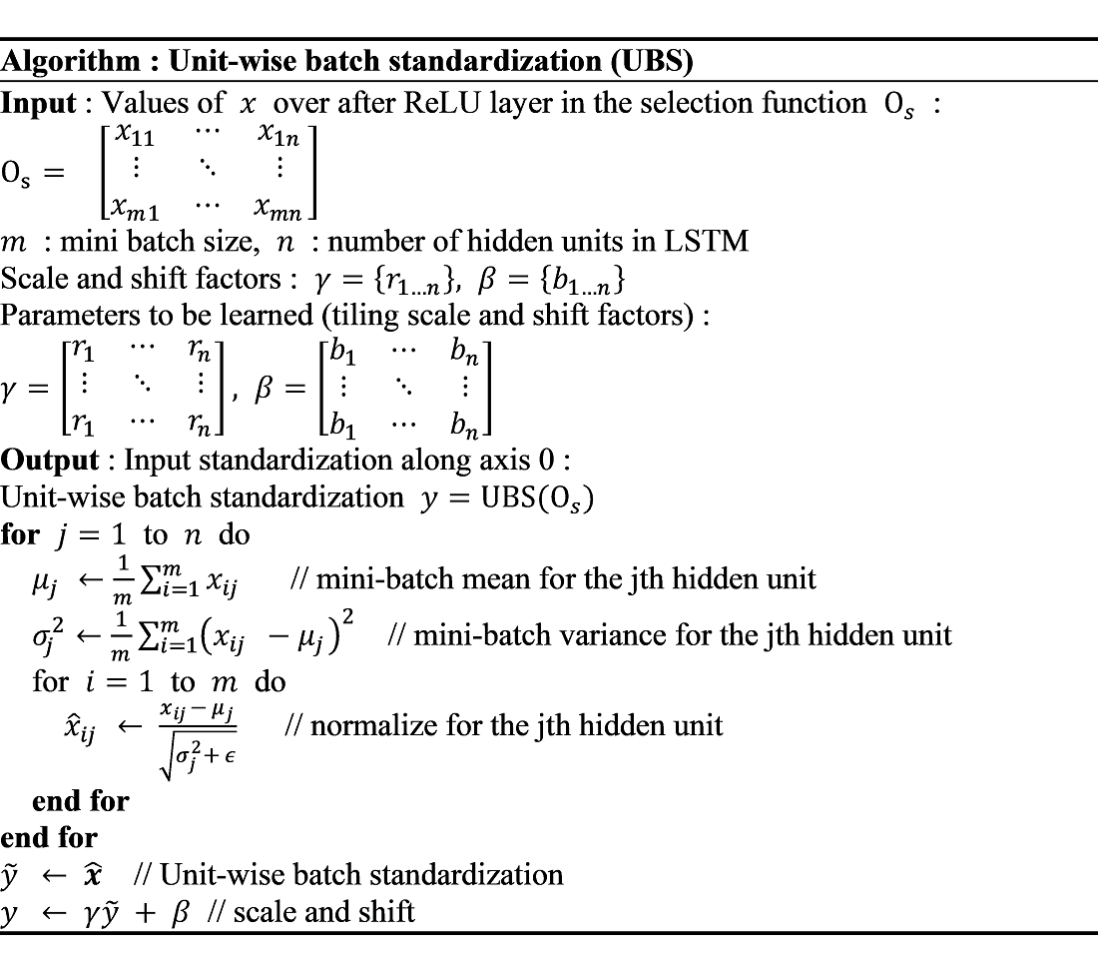
Algorithm of unit-wise batch standardization. LSTM: long short-term memory; ReLU: rectified linear unit.

In this study, a new selection function structure for LSTM models was designed. The basic frame of the selection function structure was based on a CNN-based model from a previous study [32] that used batch normalization [36] for the selection function. The detailed structure and parameters were determined through a grid search. The output shape of the *many-to-one* structure LSTM is (n_batch, n_hidden_unit), with conventional batch normalization, applying the same mean and variance to all units. However, this method of normalization ignores the features of each hidden unit in the LSTM output. To address this problem, we applied a new UBS that normalizes the batch derived from an original batch normalization [36] while preserving the hidden-unit features captured for each training sample. As shown in Table 1, UBS uses a fully connected layer that maintains the LSTM output's shape while generating the output and standardizing the batch, as shown in Figure 2. When batch normalization is applied to CNNs, normalization factors (mean and variance) are obtained from each input channel [37]. However, to preserve hidden units' individual features, we calculated normalization factors obtained from each LSTM's hidden unit.

**Table 1:** Detailed structure of the selective prediction step.

Layer	Input shape	Output shape
LSTM^a^	(n_batch, n_time steps, n_features)	(n_batch, n_hidden unit)
FC1^b,c^	(n_batch, n_hidden unit)	(n_batch, n_hidden unit)
FC2^b,d^	(n_batch, n_hidden unit)	(n_batch, n_hidden unit)
ReLU^b,e^	(n_batch, n_hidden unit)	(n_batch, n_hidden unit)
UBS^b,f^	(n_batch, n_hidden unit)	(n_batch, n_hidden unit)
FC3^g^	(n_batch, n_hidden unit)	(n_batch, 1)
Sigmoid	(n_batch, 1)	(n_batch, 1)

^a^LSTM: long short-term memory.

^b^The layer retains the input.

^c^FC1: fully connected layer 1.

^d^FC2: fully connected layer 2.

^e^ReLU: rectified linear unit.

^f^UBS: unit-wise batch standardization.

^g^FC3: fully connected layer 3.

### Performance Evaluation

In a health care system, a misdiagnosis involving a type 2 error may imply serious repercussions, and incorrect judgment involving a type 1 error may increase user fatigue. Therefore, we verified the performance of the algorithm by checking false-positive and false-negative rates. The false-positive rate (also known as type 1 error, fall-out, or false-alarm ratio) was calculated as the ratio between the number of negative events incorrectly identified as positive and the total number of actual negative events. The false-negative rate (type 2 error) was calculated as the number of samples misclassified as negative out of the total number of positive events.

### Experiment

#### Overview

##### Data Sets

This study was reviewed and approved by the institutional review board (#HYUIRB-202111-003) of the Hanyang University, and the requirement for informed consent was waived. A widely used public database was employed to verify the applicability of the selective prediction model to time series health care data. Considering that the purpose of selective prediction is to reject uncertain predictions, we selected two data sets containing classes that can be misclassified [38-42]: the *human activity recognition using smartphones* and the *Massachusetts Institute of Technology-Beth Israel Hospital* (MIT-BIH) data sets. Detailed descriptions of the data sets have been provided below.

##### Human Activity Recognition Using Smartphones Data Set

This data set consists of human gait signals monitored by an accelerometer and gyroscope with 6 different activity classes [43]. The signal was measured by attaching Samsung Galaxy S2 smartphones with embedded inertial sensors to the waists of 30 subjects aged 19 to 48 years. Each subject performed six activities (standing, sitting, laying, walking, walking upstairs, and walking downstairs) at least two times for 12 to 15 seconds. The 3-axial linear acceleration and angular velocity were measured at 50 Hz using an embedded accelerometer and gyroscope. The experiments were video-recorded to label the data manually. The signals were preprocessed using a median filter and a third-order low-pass Butterworth filter with a 20-Hz cutoff frequency and then sampled in sliding windows of 2.56 seconds with 50% overlap (128 readings/window). A total of 10,299 data points were recorded. The training data were randomly selected from 70% of the data set, and the remaining data set was used for the test. The x, y, and z components of the body accelerometer, body gyroscope, and total (gravitational and body) accelerometers were treated as 9 input features. Each sample contained 128 sequences.

##### MIT-BIH Arrhythmia Data Set

This data set contains 48 half-hour excerpts of two-channel ambulatory electrocardiogram (ECG) recordings from 47 subjects [44]. The recordings were digitized at 360 samples per second per channel with 11-bit resolution over a 10-mV range and annotated independently by 2 or more cardiologists. The data set is publicly available in the PhysioNet [45] database. All protected health information was removed and deidentified using record numbers. A method described in a previous study was used for preprocessing data [46]. First, ECG signals were divided into 10-second intervals. Subsequently, the signal was normalized between 0 and 1. Where the median of the R-R time interval in the ECG signal was T, the time from the R peak to 1.2 T was used as 1 segment. Because the length of the segment changes every 10 seconds, the length of the entire data set is zero-padded based on the longest time. The data set consisted of 109,446 data points with a sampling frequency of 125 Hz. Each data set contained 187 sequences grouped into five classes: N (normal beat), S (supraventricular premature beat), V (premature ventricular contraction), F (fusion of ventricular and normal beats), and Q (unclassifiable beat). Unclassifiable data were not included in this study. As the data for each class were highly imbalanced, 800 data samples were randomly extracted from each class [46]. The data set was sampled for every run, and result was expressed as an average of the results. The data set was then randomly divided into sets: 80% for training and 20% for testing.

### Model Architecture and Parameters Setting

#### Overview

In this study, a selective prediction model was developed using LSTM. Deep learning models such as LSTM are considered effective for extracting meaningful features from raw data. No feature extractor was used in this study because a deep learning model is suitable for use with raw data. The prediction model architecture was determined and optimized based on previous studies, and hyperparameters were optimized using an extensive grid search [47,48]. The details for each data set are described below.

#### Human Activity Recognition Using Smartphones Data Set

The LSTM model for the human activity recognition using smartphones data set had a single layer with 2 cells and 32 hidden units. For parameter setting, the learning rate was 0.0005, and the L2 regularization was set at a lambda of 0.00005. The mini batch size was 919, and the training epoch was 500. The optimal α and λ were 0.6 and 200, respectively.

#### MIT-BIH Arrhythmia Data Set

The LSTM model for the MIT-BIH arrhythmia data set had a single layer with 2 cells and 48 hidden units, a learning rate of 0.0001, a minibatch size of 640, and a training epoch of 2000. The optimal α was 0.2, and the optimal λ was 4.

### Comparison Method

To prove that the UBS is effective for developing a proper selection function in an LSTM model with a reject option, we compared it with conventional batch normalization and a model without normalization. The false-positive and false-negative rates were also calculated, and a standard LSTM model without a selection function was used as the baseline.

## Results

### LSTM Performance for Prediction

The baseline models should be optimized for LSTM models without a selection function for each data set. Therefore, we validated the LSTM model prediction performance without any selection. The test accuracies of the LSTM models optimized without a selection step for the human activity recognition using smartphones data set and the MIT-BIH arrhythmia data set are 92.35% and 97.23% for each data set. The precision of the model was 91.72% and the recall was 91.54% for the Human Activity Recognition Using Smartphones data set. For the MIT-BIH arrhythmia data set, the precision of the model was 87.13% and the recall was 78.64%. The F1-score for each data set were 91.63% and 82.67%, respectively.

### Coverage Violation

After setting the target coverage, the empirical coverage of the test set was calculated for each normalization method. The target coverage rates were obtained from a previous study [32]. As the target coverage is the target threshold, it should be set to a sufficiently reliable value. Therefore, the target coverages were set at 0.85, 0.90, and 0.95. The difference between the target coverage and the actual coverage value is called *coverage violation*, which estimates the extent to which the model can learn to select the samples as instructed by the target coverage hyperparameter. The experimental results for each data set are listed in Table 2. The coverage value was averaged for 5 different runs. As shown in Table 2, the empirical coverage with UBS produced superior results as they converged on the target coverage, whereas other normalization approaches showed relatively poor results.

**Table 2:** Empirical coverage of the human activity recognition (HAR) using smartphones and the Massachusetts Institute of Technology-Beth Israel Hospital (MIT-BIH) arrhythmia data sets by different normalization methods. Target coverage was set before training.

Target coverage	HAR using smartphones data set				MIT-BIH arrhythmia data set		
	Normalization method of selective prediction				Normalization method of selective prediction		
	UBS^a^	BN^b^	Without normalization^c^	UBS		BN	Without normalization
0.95, mean (SD)	0.9660 (0.0029)	0.9996 (0.0001)	0.9986 (0.0002)	0.9564 (0.0019)		0.9680 (0.0067)	1.0000 (0)
0.90, mean (SD)	0.9053 (0.0035)	0.9980 (0.0001)	0.9984 (0.0001)	0.9084 (0.0055)		0.9998 (0.0001)	1.0000 (0)
0.85, mean (SD)	0.8582 (0.0007)	0.9237 (0.0026)	0.9986 (0.0002)	0.8888 (0.0016)		0.9518 (0.0001)	1.0000 (0)
Average violation, %	0.98	7.38	9.85	1.79		7.32	10.00

^a^UBS: unit-wise batch standardization.

^b^BN: batch normalization (a normalization method using the mean and variance obtained from the input batch).

^c^*Without normalization* means that there was no normalization in the selection function structure.

### Selective Risk (Error Rate)

The selective risks for each normalization method are presented in Table 3. The selective risk value was averaged from 5 different runs. In the selective prediction model with LSTM, the selective risk increased with coverage. UBS normalization achieved relatively superior performance with various target coverages compared with conventional batch normalization. If normalization was not applied, the risk varied widely.

**Table 3:** Selective risk of the human activity recognition (HAR) using smartphones and the Massachusetts Institute of Technology-Beth Israel Hospital (MIT-BIH) arrhythmia data sets by different normalization methods.

Target coverage	HAR using smartphones data set				MIT-BIH arrhythmia data set		
	Normalization method of selective prediction				Normalization method of selective prediction		
	UBS^a^	BN^b^	Without normalization^c^	UBS		BN	Without normalization
0.95, mean (SD)	0.1423 (0.0041)	0.1611 (0.0445)	0.1476 (0.0068)	0.1970 (0.0038)		0.2175 (0.0108)	0.2000 (0.4472)
0.90, mean (SD)	0.1232 (0.0042)	0.1283 (0.0067)	0.1312 (0.0139)	0.1791 (0.0050)		0.3200 (0.1095)	0.2000 (0.4472)
0.85, mean (SD)	0.1136 (0.0060)	0.1170 (0.0024)	0.1267 (0.0145)	0.1585 (0.0028)		0.1967 (0.0064)	0.2000 (0.4472)
Average risk	0.1264	0.1355	0.1352	0.1782		0.2447	0.2

^a^UBS: unit-wise batch standardization.

^b^BN: batch normalization (a normalization method using the mean and variance obtained from the input batch).

^c^*Without normalization* means that there was no normalization in the selection function structure.

### False-Positive and False-Negative Rates

As the selective prediction model produced classification results only when it was confident about its own classification, we expected that both false-positive and false-negative rates would decrease. The false-positive and false-negative rates of each data set were calculated from the results of the model that achieved the best performance among 5 different runs (Tables 4 and 5). The baseline models were well-optimized LSTM models without a selection function for each data set.

**Table 4:** False-positive rates of the human activity recognition (HAR) using smartphones and the Massachusetts Institute of Technology-Beth Israel Hospital (MIT-BIH) arrhythmia data sets by different normalization methods.

Target coverage	HAR using smartphones data set					MIT-BIH arrhythmia data set				
	Normalization method of selective prediction			General prediction^a^	Normalization method of selective prediction					General prediction
	UBS^b^	BN^c^	Without normalization^d^		UBS		BN	Without normalization		
0.95, %	2.04	2.59	2.65	N/A^e^	6.34		7.67	6.93	N/A	
0.90, %	2.00	3.00	2.63	N/A	5.39		6.98	6.77	N/A	
0.85, %	2.22	3.02	2.63	N/A	5.66		7.03	7.97	N/A	
Average false-positive rate, %	2.09	2.87	2.64	2.89	5.80		7.23	7.22	6.44	

^a^General prediction is the long short-term memory classification model's false-positive rate without a selection function.

^b^UBS: unit-wise batch standardization.

^c^BN: batch normalization (a normalization method using the mean and variance obtained from the input batch).

^d^*Without normalization* means that there was no normalization in the selection function structure.

^e^N/A: not applicable.

**Table 5:** False-negative rates of the human activity recognition (HAR) using smartphones and the Massachusetts Institute of Technology-Beth Israel Hospital (MIT-BIH) arrhythmia data sets by different normalization methods.

Target coverage	HAR using smartphones data set						MIT-BIH arrhythmia data set				
	Normalization method of selective prediction				General prediction^a^		Normalization method of selective prediction				General prediction
	UBS^b^	BN^c^	Without normalization^d^			UBS		BN	Without normalization		
0.95, %	10.18	17.17	12.69	N/A^e^		18.82		23.33	20.78	N/A	
0.90, %	10.72	15.04	13.05	N/A		16.48		20.94	20.31	N/A	
0.85, %	10.85	14.46	12.94	N/A		16.41		21.44	23.91	N/A	
Average false-negative rate, %	10.58	15.56	12.89	14.48		17.24		21.90	21.67	26.47	

^a^General prediction is the long short-term memory classification model's false-positive rate without a selection function.

^b^UBS: unit-wise batch standardization.

^c^BN: batch normalization; which is a normalization method using the mean and variance obtained from the input batch.

^d^Without normalization means that there was no normalization in the selection function structure.

^e^N/A: not applicable.

### Learned Feature Representation

**Figure 3:**
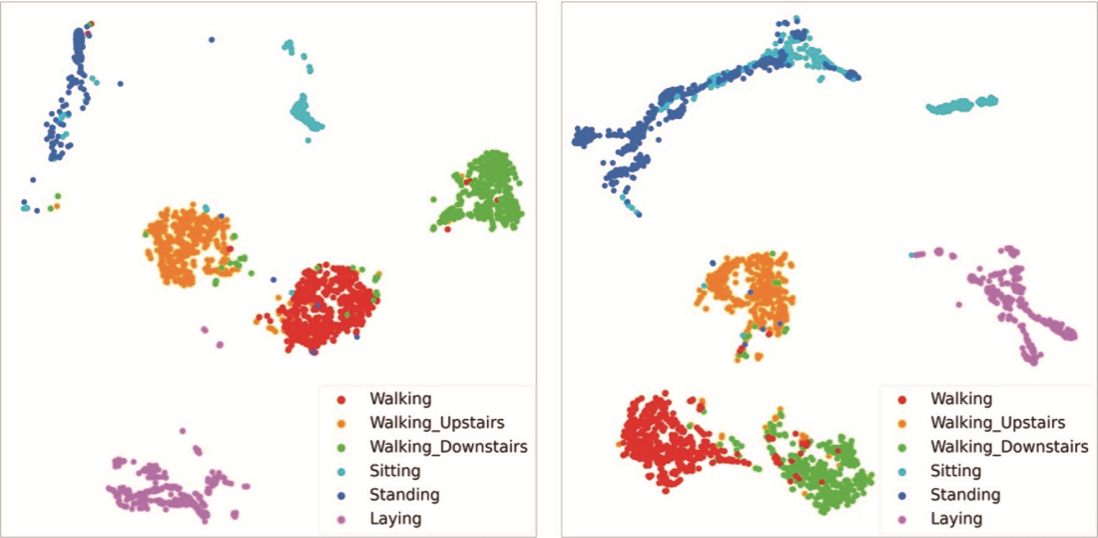
t-Distributed stochastic neighbor embedding visualizations of learned features using all test samples in the human activity recognition using smartphones data set. Left: Long short-term memory with a reject option using unit-wise batch standardization results when the target coverage was 0.95. Rejected samples were not included in this figure. Right: long short-term memory model results without a reject option.

Figure 3 shows the visualization of the features learned from the LSTM models using t-distributed stochastic neighbor embedding [49]. Figure 3 (left) depicts the test set sample that was not rejected when the target coverage was set at 0.95. The data set used in the visualization was the test set for the human activity recognition using smartphones data set. The *Sitting* (cyan) and *Standing* samples (blue) are more mixed in Figure 3 (right) than in Figure 3 (left). The *Walking_Down_Stairs* (green), *Walking_Up_Stairs* (orange), and *Walking* samples (red) are closely clustered in Figure 3 (left), whereas some of them overlap in Figure 3 (right).

## Discussion

### Principal Findings

Our objective is to develop a selective prediction model using LSTM. The developed selective prediction model rejected samples using the confidence level of classifications. This selective prediction model with a reject option was trained to determine whether to obtain a classification based on targeted coverage. If the model's classification confidence was low, the model rejected the classification and did not apply information to backpropagate on samples. As a result, the selective prediction model was trained mainly using samples that had a sufficient confidence level, which guaranteed reliability and low error rates for samples that were not rejected. To implement selective prediction for LSTM, we conducted an experiment to identify a method of normalization that could improve the performance of the selection function.

In health care systems, high accuracy is important, but low false-positive and false-negative rates are also essential. To handle various time series data obtained from a health care system, we devised a selective prediction model with LSTM using an effective selection function and focused on the structure of the function. As shown in Table 1, the output of the *many-to-one* LSTM includes hidden-unit information. Our goal was to deal with LSTMs that have *many-to-one* structures, but conventional batch normalization normalizes all batches at once. To tackle this problem, we devised UBS as a special method of normalization that attempts to normalize each hidden unit in LSTM. The false-positive and false-negative rates for each data set were meaningful. For each target coverage, the selective prediction model with UBS was superior to the model with batch normalization and the model without normalization (Tables 4 and 5). These findings show that a selective function using UBS can decrease false-positive and false-negative rates. On this basis, we interpreted that the model with UBS can learn class-specific features and consider which samples to reject in the training phase.

UBS also helped the model be trained based on target coverage and reduced selective risk. Using 2 public health data sets, the empirical coverage violation of the selective prediction was lower than that of the other 2 methods. The selection function with the UBS had the lowest selective risk (Table 3). The MIT-BIH arrhythmia data set results show that the coverage of the model without normalization was high regardless of the target coverage. These findings imply that the selective function without normalization did not perform as desired. We assumed that these results were based on whether the normalization methods considered hidden-unit characteristics of LSTM.

Regarding the learned feature representation, the classification model with the reject option differed from existing models. In Figure 3, a classification model with the reject option achieved relatively better classification performance than the conventional model without the reject option because the selective prediction LSTM model did not learn the features from samples with a low confidence level. As reported in a previous study [32], this suggests that representational capacity was not wasted because the model was trained mainly on samples with a high confidence level using selective prediction. Using this property, selective prediction allows humans to classify samples with low reliability and act as a second opinion in health care applications. In summary, the selective prediction model successfully classified samples based on high confidence-level features and simultaneously reduced the error rate by using the reject option.

Although our research supports the possibility of generating LSTM models with selective prediction, challenges remain. First, interpretation of the visualization of the learned features is limited in this study and needs to be addressed in further studies. Second, when LSTM was used for selective prediction, it was difficult to optimize parameters that control selection functions, such as α and λ, for each data set. During the experiments, we used only 2 data sets for testing and targeted only the reject option to determine the confidence level of classifications. In future studies, efficient optimization methods should be devised and applied to various models using various data sets.

### Conclusions

In this study, we developed LSTM classification models with a reject option to classify medical data time series. To develop the LSTM classification models with the reject option, UBS was applied. The UBS achieved superior performance (concerning coverage, risk, and false-positive and false-negative rates) compared with 2 other methods of normalization in experiments using 2 public time series data sets.

If the performance in classifying nonrejected samples can be maximized by adjusting coverage or selective risks, humans can trust the output of a highly confident AI model and spend more time on other rejected samples (low confidence). The final performance (human+AI) can be maximized by appropriate automation using selective prediction.

To the best of our knowledge, this is the first study demonstrating the possibility of an LSTM classification model with a reject option for time series data. Our findings may apply to various other time series data sets that require reliability.

## Abbreviations

AI: artificial intelligence
CNN: convolutional neural network
ECG: electrocardiogram
LSTM: long short-term memory
MC: Monte Carlo
MIT-BIH: Massachusetts Institute of Technology-Beth Israel Hospital
RNN: recurrent neural network
UBS: unit-wise batch standardization
